# Factors associated with physical activity in type 2 diabetes mellitus patients at a public clinic in Gaborone, Botswana, in 2017

**DOI:** 10.4102/phcfm.v11i1.2036

**Published:** 2019-08-14

**Authors:** Sunungurai Shiriyedeve, Thembelihle P. Dlungwane, Boikhutso Tlou

**Affiliations:** 1 Discipline of Public Health Medicine, School of Health Sciences, College of Health Sciences, University of KwaZulu-Natal, Durban, South Africa

**Keywords:** PAL, type 2 diabetes mellitus, burden of diabetes, Africa, Botswana

## Abstract

**Background:**

Physical activity plays a significant role in the managing of type 2 diabetes and is essential in reducing morbidity and mortality associated with diabetes mellitus. A number of factors influence non-adherence to physical activity: social, personal, environmental and economic factors. Diabetes research conducted in Botswana has focused on behavioural change, treatment adherence and nutrition. The physical activity levels of type 2 diabetes patients and associated factors are not known.

**Aim:**

The aim of this study was to assess the physical activity levels (PALs) and factors associated with physical activity in type 2 diabetes mellitus

**Setting:**

The study was conducted at a public clinic in Gaborone, Botswana, in 2017.

**Methods:**

An observational cross-sectional study was conducted at a public clinic in Gaborone, Botswana. An interview-administered questionnaire was used to assess the PALs and factors associated with physical activity in type 2 diabetes mellitus patients. Data were captured on Excel and exported to SPSS software version 25 for analysis. Chi-square test, Fischer’s exact test and Pearson’s moment correlation examined the relationship between participants’ characteristics and their engagement in regular exercise.

**Results:**

The majority of the study participants had low PALs (54.7%). The results showed a non-significant negative correlation between age and PAL (*r* = −0.085) and between sitting time (sedentary time) and PAL (−0.098).

**Conclusion:**

Most type 2 diabetes mellitus patients had low PALs. Health-promoting activities are needed to promote physical activity and thus prevent complications associated with physical inactivity.

## Introduction

According to the World Health Organization (WHO), 422 million people in the world had diabetes with an estimated worldwide prevalence of 8.5% in 2016.^1^ The number of people living with diabetes is expected to rise to 629 million by 2040 globally.^2^ The burden of type 2 diabetes mellitus is higher than type 1 diabetes mellitus, with approximately 90% to 95% of people with diabetes mellitus having type 2 diabetes mellitus.^3^

Physical activity is essential in reducing morbidity and mortality associated with diabetes mellitus and maintaining quality of life.^4^ Regular physical activity reduces high blood sugar in diabetes mellitus patients by improving the sensitivity of skeletal muscles to insulin which may result in the reduction of medication in-take.^5,6,7^ Furthermore, moderate and vigorous physical activity contributes to weight reduction, improved cardiovascular fitness and over all emotional well-being for patients living with diabetes mellitus.^5^

Physical activity plays a significant part in managing type 2 diabetes mellitus, by promoting a healthy lifestyle.^4^ Despite the reported benefits of physical activity, many individuals with diabetes mellitus fail to initiate and/or adhere to a regular WHO prescribed programme of at least 150 minutes per week of physical activity at moderate levels or 75 min of vigorous-intensity physical activity.^1^ Those patients with diabetes mellitus who exercise often discontinue within 3–6 months,^5^ with approximately 30% to 60% of the people with diabetes being reported as physically inactive.^1,6^

A number of factors influence non-adherence to physical activity, and these include social, personal, environmental and economic factors.^5,7,8^

The reasons for a decrease in physical activity among diabetes mellitus patients are because of a feeling of difficulty to exercise, tiredness and spending most of their time watching television.^9,10^ Besides, lack of time, inadequate facilities such as recreation centres and safe places to perform exercise also influence physical activity among diabetes mellitus patient.^11,12^

Physical inactivity is a major risk for development of diabetes mellitus and secondary complications. The secondary complications include retinopathy, traumatic amputations, neuropathies, nephropathy, pregnancy-related problems and hypertension.^1,13,14^ Secondary comorbidities of type 2 diabetes mellitus can result in increased direct and indirect costs to the economy.^15^ Direct costs refer to those related to hospitalisation and treatment of complications.^15^ Indirect costs are mostly related to loss of productivity because of death, illness or time spent accompanying a patient to the hospital.^16^ The International Diabetes Federation report estimated the global spending on diabetes to range between $673 billion and $1197 billion.^17^ The total annual cost of diabetes care (direct and indirect) in the sub-Saharan Africa region is approximated at $67.03bn or $ 8836bn per diabetic patient.^18^

Diabetes is sixth in the top ten causes of death in Botswana, and the WHO country profile report for 2016 revealed a diabetes prevalence of 5.6% (20–79 years) and a diabetes-related mortality of 4% (all ages) in Botswana.^19^ Diabetes research conducted in Botswana has focused on behavioural change and adherence to treatment and nutrition. The physical activity levels of type 2 diabetes mellitus patients and associated factors are not known. The aim of this study was to assess the physical activity levels (PALs) and factors associated with physical activity in type 2 diabetic patients at a public clinic in Gaborone, Botswana in 2017.

## Research methods and design

### Study design

An observational cross-sectional study design with an analytic component was implemented.

### Study setting

The study site was a public clinic in Gaborone, which is one of the two national centres of excellence for diabetes management in Botswana. The centre is managed by nurses and doctors trained in diabetes management. The clinic is an extension of the tertiary hospital. It is a catchment area for both urban and rural diabetes mellitus patients and at the same time serves as a primary health care centre for the local city residents. The clinic attends to approximately 100 diabetes patients per day. The patients come every 3 months for regular check-ups. Most of the patients who attend the clinic are in the low- to middle-income bracket as those belonging to the high-income bracket usually attend private hospitals. The clinic also serves a few expatriates.

Gaborone is the capital and commercial city of Botswana. It has a total population of 231 592 people, accounting for 11% of the total population.^20^ Gaborone covers 169 km^2^ with a population density of 370/km.^2,20^ The dominant languages spoken in Botswana, particularly in Gaborone, are Setswana and English, with a few people speaking Kalanga.

### Study population and sampling strategy

Type 2 diabetes patients aged between 18 and 65 years who attended the clinic were recruited into the study. The study was conducted from 29 May 2017 to 30 August 2017. Systematic random sampling method was used for sampling. Each day the clinic sees about 100 diabetes patients, of which approximately 80 have type 2 diabetes. The researcher took 27 patients per visit per week for the first 4 weeks and 26 patients per visit per week for the last 4 weeks of the study. Each day, the first participant was chosen at random and thereafter an interval of two (3rd person) patients was implemented to get the targeted the number. G-Power software was used for calculating the sample size, with the type 1 error being set at 0.05 and the statistical power at 80%. The critical F-value was set at 2.65 and the medium effect size was set at 0.23. The calculated sample size was 212.

### Instrument and data collections

A standardised interviewer administered questionnaire was used to assess the PALs and factors associated with physical activity in type 2 diabetic patients. The first section of the questionnaire covered socio-demographic information. The researcher adapted a validated questionnaire to assess socio-demographic status such as age, sex, number of people in the household, level of education, marital status and occupation.^4,11^ Age was categorised into three groups young, middle-aged and old-aged according to reviewed literature.^21^ Young was between 18 and 35 years, middle-aged was between 36 and 55 years and old-aged was 56+ years. The researcher added the type of medication and exercising habits to the socio-demographic section to capture all the necessary patient information. Anthropometric measures to assess nutritional status were recorded, that is, height and weight. The BMI (Body mass index) was calculated (weight [kg]/height^2^ [m^2^]) and used to rate the nutritional status according to WHO criteria. The World Health Organization classifies BMI into four categories, underweight < 18.5, normal BMI 18.5–24.9, overweight 25.0–29.9 and obese BMI ≥ 30.^22^

The second section was an adopted questionnaire from the International Physical Activity Committee (IPAQ), which measured the PAL.^23^ A separate IPAQ question assessed the time spent sitting to measure sedentary activity but was not part of the total physical activity score.^24^ It assessed the time spent sitting on a typical day, and weekend day, and the time spent sitting during travel.^24^ The last section covered the barriers and motivators to physical activity as reported by the patient.^11,25^

The questionnaire was pretested with 13 patients to ensure that it was user-friendly. The questionnaire was translated into Setswana by a qualified translator. The translations were back translated into English to ensure that they are compatible with the original questionnaire. Permission to conduct the study was obtained from the hospital manager. Qualified nurses screened the patients and those that met the inclusion criteria and agreed to participate were referred to the research assistant.

### Data analysis

The data were captured into Microsoft Excel and then exported into SPSS version 25. Descriptive statistics were summarised using frequencies, while chi-square and Fischer’s exact statistics were used to determine the associations between participant’s characteristics and PAL. Where the cell frequencies are less than 5, the Fischer’s exact test is used. The Pearson’s product-moment coefficient was calculated to find the correlation between PAL and participant’s characteristics. A *p*-value < 0.05 was considered to be statistically significant.

The level of physical activity was analysed by taking into consideration the metabolic equivalent of task (MET)-minutes per week indicators and the days of physical activity. To calculate MET-minutes per week per participant, the following formula was used: (MET value) × (time of activity in minutes per day) × (days of activity per week) = MET-minutes per week. The overall PAL was categorised as high, moderate or low. High PAL refers to seven or more days of any combination of walking, moderate-intensity or vigorous-intensity activities achieving a minimum total physical activity of at least 3000 MET-minutes per week.^24^ For moderate PAL, the criteria are five or more days of any combination of walking, moderate-intensity or vigorous-intensity activities, and achieving a minimum total physical activity of at least 600 MET-minutes per week.^24^ Low PAL is the lowest level of physical activity and is below 600 MET per week.

### Ethical considerations

Ethical clearance was obtained from the University of KwaZulu-Natal Biomedical Research Ethics Committee (BE540/16). Permission to conduct the study was obtained from the Hospital Ethics Committee (PMH 5/79(286-4-2017)) and from the Botswana Ministry of Health (HPDME 13/18/1(863)). All patients who agreed to participate in the study signed an informed consent form. Questionnaires and signed consent forms were filed separately to ensure anonymity of the questionnaire. Participation was voluntary, and participants were informed that they could withdraw from the study without prejudice. The anonymous questionnaires were labelled with unique numbers to ensure confidentiality.

## Results

One hundred and seventy patients participated in the study. Table 1 shows the socio-demographic data of the participants. The average age of the participants was 51 (SD 13.3) with the majority being females 65.7% and in the 56+ age group (47.6%). Most of the participants were single or widowed or divorced (60%), 58% had secondary education and 47.6% were not-employed. In the study, 61.2% of the participants reported to be staying in urban areas while 38.8% in rural areas. Most of the study population were obese (58.1%) (Table 1).

**TABLE 1:** Participant’s socio-demographic profile.

Variable	*N*	%
**Sex**		
Female	111	65.7
Male	58	34.3
**Age (mean 51, SD 13.3)**		
18–35	29	17.3
36–55	59	35.1
56+	80	47.6
**Marital status**		
Single	68	40.0
Married	68	40.0
Divorced	8	4.7
Widowed	26	15.3
**Level of education**		
No formal	22	22.0
Primary	40	40.0
Secondary	58	58.0
Tertiary	44	44.0
**Employment status**		
Employed	55	32.4
Unemployed	81	47.6
Self-employed	30	17.6
Student	4	2.4
**Dwelling place**		
Rural	62	38.8
Urban	98	61.2
**BMI**		
Underweight	3	1.9
Normal	21	13.1
Overweight	43	26.9
Obese	93	58.1

*Source*: Adapted from Shazwani and Parajuli.^4,11^

More than half of the participants had low PAL (54.7%) with only 14 participants (8.2%) having high PAL (Table 2). The revealed average sitting time of the respondent was 5.8 h per day in the past week (SD: 5.4).

**TABLE 2:** Levels of physical activity.

PAL	Frequency	%	Valid %
Low	93	54.7	54.7
Moderate	63	37.1	37.1
High	14	8.2	8.2
**Total**	**170**	**100.0**	**100.0**

PAL, physical activity levels.

The study went on to assess the association between socio-demographics and PAL. There was a weak negative correlation between age and PAL (*r* = −0.085) and between sitting time (sedentary time) and PAL (−0.098). There was no statistical significant association between PAL and the following socio-demographic factors: sex, marital status, level of education, employment status, dwelling place and BMI (Table 3).

**TABLE 3:** Relationship between physical activity and socio-demographic.

Variables	Physical activity						*p*
	Low		Moderate		High		
	*N*	%	*N*	%	*n*	%
**Age (years)**							
18–35	14	48.3	13	44.8	2	6.9	0.069
36–55	30	50.8	21	35.6	8	13.6	
56+	48	60.0	28	35.0	4	5.0	
**Gender**							
Male	32	55.2	20	34.5	6	10.3	0.751
Female	60	54.5	42	38.2	8	7.3	
**Marital status**							
Single	32	47.1	31	45.6	5	7.4	0.553
Married	39	57.4	22	32.4	7	10.3	
Divorced	5	62.5	2	25.0	1	12.5	
Widowed	17	65.4	8	30.8	1	3.8	
**Employment**							
Employed	23	41.8	25	45.5	7	12.7	0.117
Unemployed	53	65.4	24	29.6	4	4.9	
Self-employed	14	46.7	13	43.3	3	10.0	
Student	3	75.0	1	25.0	0	0.0	
**Level of education**							
No formal education	14	63.6	7	31.8	1	4.5	0.599
Primary	22	55.0	16	40.0	2	5.0	
Secondary	26	44.8	26	44.8	6	10.3	
Tertiary	27	61.4	13	29.5	4	9.1	
**Nature of dwelling**							
Rural	34	54.8	21	33.9	7	11.3	0.689
Urban	55	56.1	36	36.7	7	7.1	
**Involved in exercise**							
Yes	62	52.5	46	39.0	10	8.5	0.739
No	30	58.8	17	33.3	4	7.8	
**Exercising partner**							
Alone	42	55.3	29	38.2	5	6.6	0.545
Spouse	3	50.0	3	50.0	0	0.0	
Friend/family	14	42.4	14	42.4	5	15.2	
Gym	3	100.0	0	0.0	0	0.0	
**Body mass index (BMI)**							
Underweight	0	0.0	1	100.0	0	0.0	0.818
Normal	8	50.0	7	43.8	1	6.3	
Overweight	21	60.0	11	31.4	3	8.6	
Obese	37	49	31	40.8	8	10.5	

Participant’s motivation and barriers to exercise were identified. Most participants were highly motivated to engage in physical activity by wanting to be healthy 73.3%, wanting to look good 66.9%, liking exercise 57.1% and wanting to lose weight 50.3% (Table 4). The most reported barriers were no place to exercise (8.8%) and no one to exercise with (8.7%) (Table 5).

**TABLE 4:** Motivators to regular physical activity.

Variable	Not a motivator (%)	Slight motivator (%)	Moderate motivator (%)	Major motivator (%)
I want to be healthy	15.8	6.7	4.2	73.3
I was told to exercise	21.3	9.8	19.5	49.4
I have someone to exercise with	60.0	12.1	4.2	23.6
I having gym money	74.2	11.0	3.7	11.0
I have time to exercise	29.6	12.3	18.5	39.5
I like exercising	24.5	8.6	9.8	57.1
I want to lose weight	31.9	6.7	11.0	50.3
I want to looking good	22.7	4.3	6.1	66.9
Other	67.7	7.5	4.3	20.5

*Source*: Adapted from Shazwani and Sjors.^11, 25^

**TABLE 5:** Barriers to physical activity.

Variable	Not a barrier (%)	Slight barrier (%)	Moderate barrier (%)	Major barrier (%)
I have health problems	75.3	13.9	4.4	6.3
I don’t have time	81.9	5.6	5.6	6.9
I have no one to exercise with	73.9	9.3	8.1	8.7
I have no to place to exercise	74.2	10.7	6.3	8.8
Unsafe to exercise	85.0	5	.0	6.3
I was not told about the importance of exercise	75.7	11.2	9.2	4.0
I get hypoglycaemic	87.5	6.3	3.1	3.1
I do not like exercising	95.6	1.9	0.6	1.9
I have no one to look after children	88.1	5.6	3.1	3.1
Other	95.6	1.9	0.6	1.9

*Source*: Adapted from Shazwani and Sjors.^11, 25^

## Discussion

This study assessed PALs and the factors associated with physical activity in type 2 diabetes mellitus patients at a clinic in Botswana. Physical activity is a chief cornerstone in the management of type 2 diabetes although diabetes patients still remain inactive.^1,4,5^ The findings of this study indicated that majority of the participants had low PAL (54.7%). The results are similar to other studies that used the short IPAQ form although different to another study that used the long IPAQ form like in this study. Low PAL was found in Western Nigerians (62%) and in a medically underserved community in America (62.9%) with type 2 diabetes mellitus.^26,27^ The study that used a long form found 31% of type diabetes mellitus patients to be physical inactivity.^28^

The average sitting time (sedentary) of the participants in the study was 5.8 hours per day (SD: 5.4) in the past week. Similar results were reported in Nigeria in which type 2 diabetes patients were sedentary for a minimum of 4.8 h per day in the past week.^28^ A study conducted in America also reported a similar trend.^27^ Type 2 diabetes patients were shown to be sedentary for a minimum of 5.8 h per day (SD 4.5) in the past week.^27^

In this study, PAL was weakly and negatively related to sedentary lifestyle (*r* = −0.098). The results are similar to those of a study conducted in Malaysia which reported a weak and negative relationship between PAL and sedentary lifestyle (*r* = −0.175) among diabetes patients.^11^

In this study, there was a weak negative correlation between age and PALs, as diabetes patients got older their PALs decreased. The older patients were shown to have low PALs. Similar patterns have been found in other studies, where older diabetes mellitus patients were found to have lower PALs.^9,23,29,30^ This is consistent with the results found in Yemen 2017, where type 2 diabetes patients who were younger were found to be about four times more likely to adhere to exercise programmes than those older.^29^ This might be because of the increase in the diabetes mellitus complications, degenerative changes and/or the decline in motor function as people get older leading to low physical activity.^4,29, 31,32,33^ However the results in this study show that health problems were not considered a common barrier to physical activity by most of the participants. Further reason for the low PAL in older type 2 diabetes mellitus patients might be that older patients are retired from active employment, thus generally decreasing their PAL.^8^

There was no statistically significant relationship between employment status and PAL, although most of the unemployed (65%) had low PAL. The results in this study are in contrast to the results found in other studies, employed type 2 diabetes mellitus patients were shown to be significantly more physically active than their unemployed counter parts either intentionally or incidentally.^8,10^ The reason for the increased PAL in employed diabetes mellitus patients might be that employed people tend to socialise, walk briskly to achieve tasks and do other work-related activities.^8,10^ Skilled and semi-skilled diabetes mellitus patients were more active in daily routine work as compared to those categories of occupation.^8^ High levels of education have been associated with a satisfactory understanding of the diabetes mellitus management which subsequently increases PAL.^28,33^ Highly educated people may be economically more stable than their uneducated counterparts increasing their chances of accessing and affording exercising space.^4^ However, this study did not find significant association between educational level and PAL.

Low PALs were reported mostly among the obese (50%) and overweight (60%) participants in this study. However, this study did not find any significant association between BMI and PAL (*p* > 0.05). The findings of this study concur with the results of the study in Malaysia, which did not find any significant association between BMI and PAL.^11^ In contrast, studies in Ireland and America found that those with normal BMI participate more in physical activities than those with high BMI.^27,34^ This could be attributed to the barriers mentioned by those with high BMI such as physical discomfort and embarrassment.^9,34^ On the contrary, some studies have revealed that those with high BMI have high PAL because of their understanding of the effect of weight on diabetes mellitus.^4,7^

Social support in the form of a partner or spouse plays a major role in patient’s decision to engage in physical activity.^4^ Married people tend to engage in physical activity as they have the support of their partners and families.^4,8,11,35^ This study revealed that ‘no one to exercise with’ had the highest score among the barriers to PAL. However, this study did not find any statistically significant association between marital status and PAL (*p* > 0.05). This is in contrast with studies that reported that married people have higher levels of physical activity as compared to their single, widowed and divorced counterparts.^4,8,11,35^

Men have a higher physical activity level compared to their female counterparts.^7,11,33^ The same pattern has been found in the general population.^36^ The difference in PAL between men and women might be because of the difference in social obligations between them; men are expected to be masculine as breadwinners, while women are usually restricted around the house.^28,33^ This study did not find any significant association between gender and PAL.

Environmental factors such as dwelling place has an effect on physical activity.^9,28^ A number of urban people have cars and tend to lead a more sedentary lifestyle, while in the rural areas people are more involved in activities that require manual work such as farming.^9^ In this study, a larger proportion of both urban (56.1%) and rural (54.8%) dwellers had low PALs. There was no association identified between dwelling place and PAL in this study. Similarly, a study in Ireland, Western Europe, did not find any significant relationship between PAL and dwelling place.^34^

In this study, the common motivators to physical activity were the need to be healthy (73.3%), wanting to look good (66.9%), liking to exercise (57.1%) and wanting to lose weight (50.3%). These findings are comparable to a Malaysian study that reported the perceived motivators to exercise being: the willingness to be healthy, a realisation of the importance of exercise, love of exercise and daily routine, with the willingness to exercise having the highest score.^11^ Lack of time, health problems, discomfort, fear of hypoglycaemia, laziness, cost of gym and unsafe environments are perceived barriers for physical activity among diabetes mellitus patients.^8,9,11,32,33,34,37^ This study found that the absence of exercising space and no one to exercise with were the common reported barriers to physical activity.

## Study limitations

The reliance on self-reporting data is a limitation of the study as participants may have over- or under-reported in the questionnaire which is prone to social desirability bias. The study was conducted in one clinic and the results may therefore not be generalisable to patients in other clinics. The study failed to reach the desired minimum sample size because of fluctuations in the number of patients seen per day within the 3 months study period decreasing the study power and increasing the margin error. Some variables were also missing completely at random which might have affected the results.

Further studies need to be conducted on adherence to physical activity after demonstration and giving type 2 diabetes mellitus patients exercise programmes. Objective measures such accelerometers should be incorporated into the assessment of PALs to reduce social desirability bias found in self-report PALs. Case-control studies comparing the PALs of patients with diabetes mellitus and those without diabetes need to be performed. A qualitative study to get an in-depth understanding of the highlighted barriers and motivators to physical activity is needed.

## Recommendations

Physical activity needs to be incorporated in the assessment of type 2 diabetes mellitus patients and should be consistently evaluated in the same way as glycaemic control. A multi-disciplinary approach to diabetic management should consistently encourage and emphasise the importance of physical activity in the management of type 2 diabetes mellitus. There is necessity for a resident physiotherapist, or exercise specialist, who can tailor patient-specific exercises, the same way dieticians create patient-specific diets.

## Conclusion

This study revealed that type 2 diabetes mellitus patients at a public clinic in Gaborone have low PALs. The desire to look good and liking exercise were the common motivators to physical activity. Absence of exercising space and no one to exercise with were the common reported barriers to physical activity. Health-promoting activities are recommended to promote physical activity, especially in the older patients, and thus prevent complications associated with physical inactivity among patients with type 2 diabetes mellitus.

## References

[1] World Health Organization Global report on diabetes – World Health Organization. Report No.: 9789241565257 WHO; 2016.

[2] International Diabetes Federation International Diabetes Federation Diabetes Atlas. 8th ed. Brussels, Belgium: International Diabetes Federation; 2017.

[3] Nyenwe EA, Odia OJ, Ihekwaba AE, Ojule A, Babatunde S Type 2 diabetes in adult Nigerians: A study of its prevalence and risk factors in Port Harcourt, Nigeria. Diabetes Res Clin Pract. 2003;62(3):177–185. 10.1016/j.diabres.2003.07.002 14625132

[4] Parajuli J, Saleh F, Thapa N, Ali L Factors associated with nonadherence to diet and physical activity among nepalese type 2 diabetes patients; a cross sectional study. BMC Res Notes. 2014;7(1):758–767. 10.1186/1756-0500-7-758 25344089 PMC4230343

[5] Sigal RJ, Kenny GP, Wasserman DH, Castaneda-Sceppa C Physical activity/exercise and type 2 diabetes. Diabetes Care. 2004;27(10):2518–2539. 10.2337/diacare.27.10.2518 15451933

[6] Çolak TK, Acar G, Dereli EE, et al Association between the physical activity level and the quality of life of patients with type 2 diabetes mellitus. J Phys Ther Sci. 2016;28(1):142–147. 10.1589/jpts.28.142 26957746 PMC4755992

[7] Sieńko-Awieraniów E, Stępień-Słodkowska M, Latkowska A, Głowacka E Factors Associated with physical activity in people with diabetes. Cent Eur J Sport Sci Med. 2015;12(4):73–81. 10.18276/cej.2015.4-08

[8] Jadawala H, Pawar A, Partel P, et al Factors associated with nonadherence to diet and physical activity among diabets patients: A cross sectional study. Ntl J Commun Med. 2017;8(2):68–73.

[9] Kabanda A, Phillips J Physical activity among adults with diabetes mellitus in Rwanda. Sahara J. 2011;17(2):239–247. 10.4314/ajpherd.v17i2.67663

[10] Murano I, Asakawa Y, Mizukami M, et al Factors increasing physical activity levels in diabetes mellitus: A survey of patients after an inpatient diabetes education program. J Phys Ther Sci. 2014;26(5):695–699. 10.1589/jpts.26.695 24926134 PMC4047234

[11] Shazwani N, Suzana S, Hanis Mastura Y, et al Assessment of physical activity level among individuals with type 2 diabetes mellitus at Cheras Health Clinic, Kuala Lumpur. Malays J Nutr. 2010;16(1):101–112. 22691857

[12] College TPaFoH Obesity prevention source: Environmental barriers to activity [homepage on the Internet]. Harvard College; 2017 [cited]. Available from: http://www.hsph.harvard.edu/obesity-prevention-souurce/obsity-cuases/physical-activity-environment/.

[13] Linnenkamp U, Guariguata L, Beagley J, Whiting D, Cho N The International Diabetes Federation Diabetes Atlas methodology for estimating global prevalence of hyperglycaemia in pregnancy. Diabetes Res Clin Pract. 2014;103(2):186–196. 10.1016/j.diabres.2013.11.004 24300016

[14] International Diabetes Federation International Diabetes Federation Diabetes Atlas [homepage on the Internet]. 6th ed. Brussels, Belgium: International Diabetes Federation; 2013 [cited 2016 Mar 18]. Available from: www.idf.org/membership/afr/botswana.

[15] Stellefson M The chronic care model and diabetes management in US primary care settings: A systematic review. Prev Chronic Dis. 2013;10:26 10.5888/pcd10.120180 PMC3604796 23428085

[16] International Diabetes Federation International Diabetes Federation Diabetes Atlas. 7th ed. Brussels, Belgium: International Diabetes Federation; 2015.

[17] International Diabetes Federation International Diabetes Federation Diabetes Atlas. 6th ed. Brussels, Belgium: International Diabetes Federation; 2015.

[18] Hall V, Thomsen RW, Henriksen O, Lohse N Diabetes in Sub Saharan Africa 1999–2011: Epidemiology and public health implications. A systematic review. BMC Public Health. 2011;11(1):11–564. 10.1186/1471-2458-11-564 21756350 PMC3156766

[19] World Health Organization Noncommunicable disease country profiles, 2014. Geneva: WHO; 2014.

[20] Botswana Statistics Population and Housing Census, 2011. Analytical Report Gaborone; 2011 ISBN 978-99968-428-2-5.

[21] Petry NM A comparison of young, middle-aged, and older adult treatment-seeking pathological gamblers. Gerontologist. 2002;42(1):92–99. 10.1093/geront/42.1.92 11815703

[22] World Health Organization Obesity and overweight factsheet [Online]. Geneva; 2011 Contract No.: N 311.

[23] Hallal PC, Andersen LB, Bull FC, et al Global physical activity levels: Surveillance progress, pitfalls, and prospects. Lancet. 2012;380(9838):247–257. 10.1016/S0140-6736(12)60646-1 22818937

[24] Committee IR Guidelines for data processing and analysis of the International Physical Activity Questionnaire (IPAQ)–short and long forms. 2005 [cited]. Available from: https://sites.google.com/site/theipaq/.

[25] Sjörs C, Bonn SE, Lagerros YT, Sjölander A, Bälter K Perceived reasons, incentives, and barriers to physical activity in Swedish elderly men. Interact J Med Res. 2014;3(4):215–221. 10.2196/ijmr.3191 PMC4285757 25488655

[26] Oguntibeju O, Odunaiya N, Oladipo B, Truter E Health behaviour and quality of life of patients with type 2 diabetes attending selected hospitals in south western Nigeria. West Indian Med J. 2012;61(6):619–626. 23441358

[27] Cooper J, Stetson B, Bonner J, et al Self-reported physical activity in medSically underserved adults with type 2 diabetes in clinical and community settings. J Phys Act Health. 2015;12(7):968–975. 10.1123/jpah.2013-0475 25154022

[28] Oyewole OO, Odusan O, Oritogun KS, Idowu AO Physical activity among type-2 diabetic adult Nigerians. Ann Intern Med. 2014;13(4):189–194. 10.4103/1596-3519.142290 25287033

[29] Alhariri A, Daud F, Saghir S Factors associated with adherence to diet and exercise among type 2 diabetic patients. Open Med. 2017;7(3):264–271. 10.1155/2017/1273084

[30] Alwan A Global status report on noncommunicable diseases 2010. Report No.: 9241564229 Geneva: World Health Organization; 2011.

[31] World Health Organization Diabetes country profiles, 2016 [homepage on the Internet]. 2016 [cited 2017 Oct 2]. Available from: www.who.int/entity/diabetes/country-profiles/bwa_en.pdf?ua=1

[32] Dutton GR, Johnson J, Whitehead D, Bodenlos JS, Brantley PJ Barriers to physical activity among predominantly low-income African-American patients with type 2 diabetes. Diabetes Care. 2005;28(5):1209–1210. 10.2337/diacare.28.5.1209 15855592

[33] Lawton J, Ahmad N, Hanna L, Douglas M, Hallowell N ‘I can’t do any serious exercise’: Barriers to physical activity amongst people of Pakistani and Indian origin with type 2 diabetes. Health Edu Res. 2006;21(1):43–54. 10.1093/her/cyh042 15955792

[34] Egan A, Mahmood W, Fenton R, et al Barriers to exercise in obese patients with type 2 diabetes. QJM. 2013;106(7):635–638. 10.1093/qjmed/hct075 23525164

[35] Katzmarzyk PT, Church TS, Janssen I, Ross R, Blair SN Metabolic syndrome, obesity, and mortality. Diabetes Care. 2005;28(2):391–397. 10.2337/diacare.28.2.391 15677798

[36] Joubert J, Norman R, Lambert EV, et al Estimating the burden of disease attributable to physical inactivity in South Africa in 2000: Original article. S Afr Med J. 2007;97(8):725–731. 17952230

[37] Duarte CK, Almeida JCD, Merker AJS, Brauer FdO, Rodrigues TDC Physical activity level and exercise in patients with diabetes mellitus. Rev Assoc Med Bras. 2012;58(2):215–221. 10.1016/S0104-4230(12)70183-5 22569617

